# Edible Cyanobacterial Genus *Arthrospira*: Actual State of the Art in Cultivation Methods, Genetics, and Application in Medicine

**DOI:** 10.3389/fmicb.2017.02541

**Published:** 2017-12-18

**Authors:** Magda A. Furmaniak, Agnieszka E. Misztak, Martyna D. Franczuk, Annick Wilmotte, Małgorzata Waleron, Krzysztof F. Waleron

**Affiliations:** ^1^Chair and Department of Pharmaceutical Microbiology, Medical University of Gdańsk, Gdańsk, Poland; ^2^Department of Biotechnology, Intercollegiate Faculty of Biotechnology of University of Gdańsk and Medical University of Gdańsk, Gdańsk, Poland; ^3^InBios-Centre for Protein Engineering, Department of Life Sciences, University of Liège, Liège, Belgium

**Keywords:** *Arthrospira*, Spirulina, genetics, biotechnology, medical application, cyanobacteria

## Abstract

The cyanobacterial genus *Arthrospira* appears very conserved and has been divided into five main genetic clusters on the basis of molecular taxonomy markers. Genetic studies of seven *Arthrospira* strains, including genome sequencing, have enabled a better understanding of those photosynthetic prokaryotes. Even though genetic manipulations have not yet been performed with success, many genomic and proteomic features such as stress adaptation, nitrogen fixation, or biofuel production have been characterized. Many of above-mentioned studies aimed to optimize the cultivation conditions. Factors like the light intensity and quality, the nitrogen source, or different modes of growth (auto-, hetero-, or mixotrophic) have been studied in detail. The scaling-up of the biomass production using photobioreactors, either closed or open, was also investigated to increase the production of useful compounds. The richness of nutrients contained in the genus *Arthrospira* can be used for promising applications in the biomedical domain. Ingredients such as the calcium spirulan, immulina, C-phycocyanin, and γ-linolenic acid (GLA) show a strong biological activity. Recently, its use in the fight against cancer cells was documented in many publications. The health-promoting action of “Spirulina” has been demonstrated in the case of cardiovascular diseases and age-related conditions. Some compounds also have potent immunomodulatory properties, promoting the growth of beneficial gut microflora, acting as antimicrobial and antiviral. Products derived from *Arthrospira* were shown to successfully replace biomaterial scaffolds in regenerative medicine. Supplementation with the cyanobacterium also improves the health of livestock and quality of the products of animal origin. They were also used in cosmetic preparations.

## Background

*Arthrospira* is an extremophilic pioneer organism with optimal growth temperatures around 35°C. Therefore, it is most abundant in tropical and subtropical regions. Ancient civilizations like the Aztecs were first to recognize the nutritional value of these organisms. Inhabitants of Europe came into contact with *Arthrospira* during the colonization of America in the sixteenth century, hence the first references made at that time (Ciferri, 1983). Nowadays, it is still harvested, dried, and consumed by the Kanembu tribe in Chad (Africa) and called “dihé.” Scientific interest in *Arthrospira* species was boosted after the first chemical analyses of biomass from dihé in late 1960's (Ciferri, 1983). Subsequently, it started to be extensively studied due to its nutritional properties.

**Figure 1:**
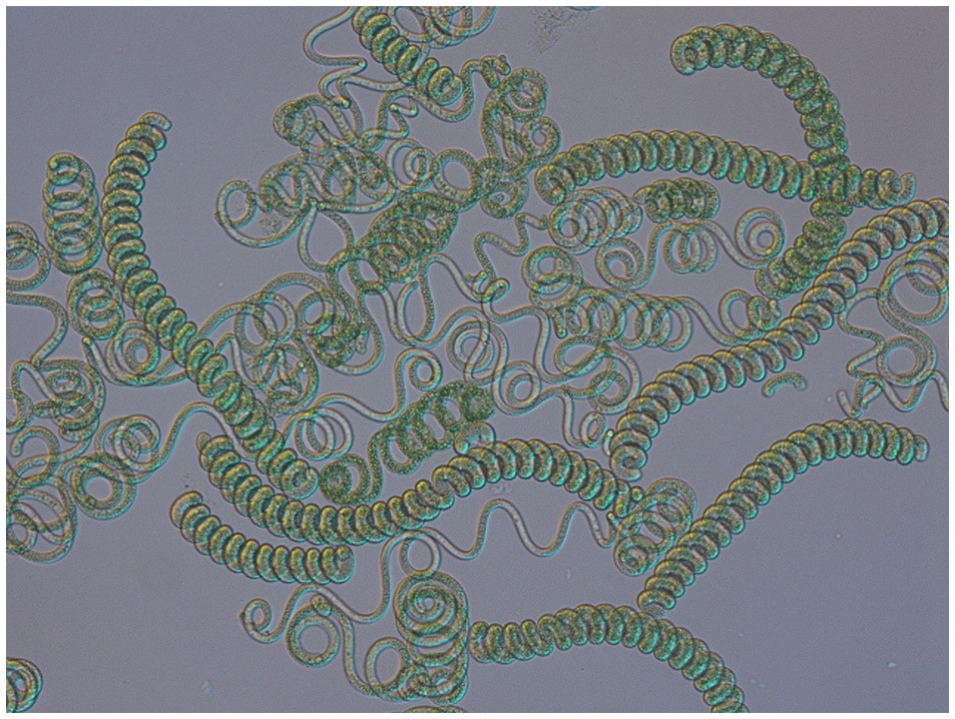
Light microscopy of *Arthrospira* filaments from natural environment (magnification 200x).

*Arthrospira* is a genus of the cyanobacterial phylum. This multicellular organism is characterized by open helical trichomes that give it a typical morphology and it has a recognized biotechnological potential (Figure 1). The oldest reference for the consumption of *Arthrospira* dates back from the sixteenth century, but its use could even be more ancient (Miklaszewska et al., 2008b). Since the genus was rediscovered in Chad and Mexico in the 1950's, the interest for *Arthrospira* applications has grown and diversified from nutrition to health, and biotechnology. However, until now, genetic engineering has turned out to be impossible due to the lack of proper technology. Fortunately, recent technological advances in the sequencing of entire genomes and genetic studies have shed a new light on its basic molecular biology as well as cyanobacterial genetics.

One of the most promising projects including *Arthrospira* is the Micro-Ecological Life Support System Alternative (MELiSSA) that aims to create an artificial ecosystem to recycle waste and oxygen for long-duration space expeditions (Gòdia et al., 2002). This multinational project consists of four compartments utilizing microbial consortia (compartment 1) or defined microorganisms (compartments 2–4). The four compartments form a closed loop that is fed by the wastes from the human crew and vegetable left-overs to recycle them. The effluent from one compartment becomes the influent of the next one. The last compartment is responsible for the removal of CO_2_ from atmosphere, water recycling, and production of food and oxygen. It includes both higher plants and the *Arthrospira* strain PCC8005 (Hendrickx et al., 2006; Badri et al., 2015).

**Figure 2:**
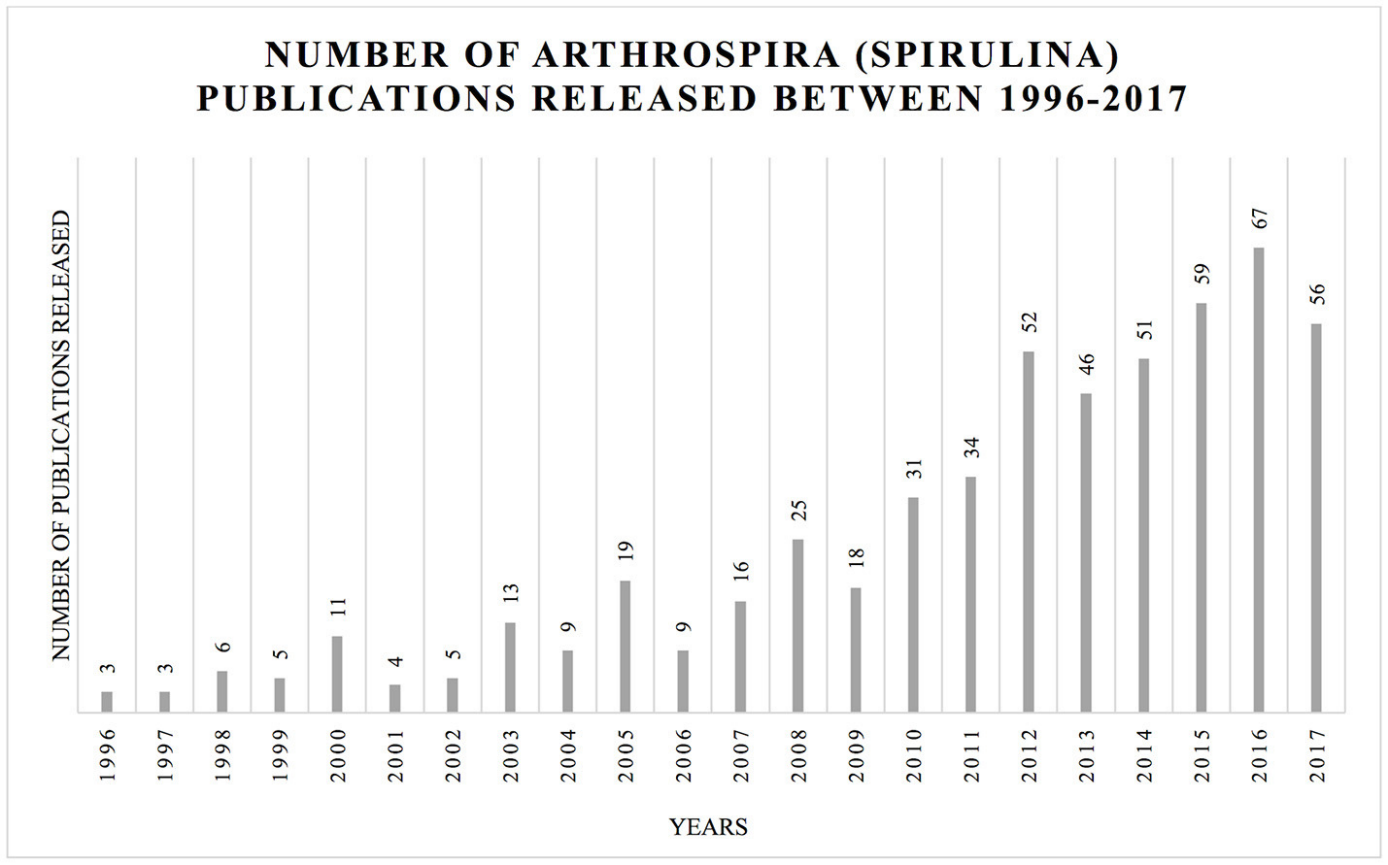
Increasing number of publications in the topic of *Arthrospira* (Spirulina) between 1996 and October 2017. The histogram illustrates the number of hits for *Arthrospira* (Spirulina) using Web of Science (https://apps.webofknowledge.com) platform using respective keywords: TOPIC: (Arthrospira) *AND* TOPIC: (Spirulina platensis); Timpespan: All years.

The last comprehensive overview dedicated to *Arthrospira* was published in 1997 (Vonshak). As a result of the scientific interest in this taxon, a large amount of data has been generated (Figure 2). Here, we summarize the information crucial for the understanding of the modern biology and perspectives concerning this genus.

## Genetics

The DNA content of the dry biomass of *Arthrospira* varies from 0.6 to 1%, which is similar to other unicellular cyanobacteria, but significantly lower than other bacteria or yeast (4–10%). The guanidine plus cytosine content is in the range 44–45 mol%. The average size of the *Arthrospira* genomes is 6.1 Mbp (Ciferri, 1983; Fujisawa et al., 2010; Cheevadhanarak et al., 2012; Lefort et al., 2014). In addition, the biomass of *Arthrospira* is rich in substances such as polysaccharides and polyphenols that are very difficult to remove during the DNA isolation process (De Philippis and Vincenzini, 1998; Morin et al., 2010). Apart from the low content of nucleic acids in its dry biomass, *Arthrospira* genomes seem to lack non-chromosomal DNA. The last report concerning the isolation of plasmid from *Arthrospira* was published in 1993 (Song et al., 1993), and has not been reproduced. These missing plasmids would have been indispensable tools for gene manipulation.

### Phylogenetics

The commercial name of *Arthrospira* products—“Spirulina”—causes a constant confusion. In 1932, Geitler has merged the genera *Arthrospira* and *Spirulina* (Geitler, 1932). The new name was *Spirulina* because this genus had been described by Turpin a few years before Stizenberger described *Arthrospira* (Vonshak, 1997). Research carried out in the last two decades showed that at ultrastructure and genetic (percentage of G+C, 16S rRNA gene sequence) level, *Arthrospira* and *Spirulina* were very distinct organisms (Herdman et al., 1979; Guglielmi et al., 1993; Nelissen et al., 1994). Chemotaxonomically, those two genera were also shown to be different, as the *Arthrospira* fatty acids profile included γ-linolenic acid (GLA), that is absent in *Spirulina* (Cohen and Vonshak, 1991). A phylogenetic study based on the *cpcA-cpcB* locus revealed that the closest relative of the *Arthrospira* genus is *Planktothrix* and that it is not related with *Spirulina* (Manen and Falquet, 2002). The study by Shih et al. (2013) presents an analysis of 74 publicly available and 54 newly sequenced cyanobacterial genomes, including 4 belonging to *Arthrospira*. The strains used in this study were diverse at the phylogenetic and phenotypical level. According to Shih et al. (2013), *Lyngbya* was the closest relative of *Arthrospira* based on 16S rRNA sequence, however, the *Planktothrix* genus was not included in this database.

The *Arthrospira* strains show high morphological similarities but also genetic homogeneity. This complicates their taxonomy, that is the subject of a few publications (Desikachary and Jeeji Bai, 1992; Komárek and Anagnostidis, 2005). At the morphological level, scientists have observed spontaneous changes from a spiral to a straight morphology in *Arthrospira* trichomes, dependent on cultivation conditions (Desikachary and Jeeji Bai, 1996; Tomaselli, 1997; Muhling et al., 2005; Wang and Zhao, 2005).

The ITS (Internally Transcribed Sequence) is a non-functional spacer situated between structural rRNA genes in the precursor transcript, and is subjected to less conservation pressures than the structural genes. It is used in molecular taxonomy to distinguish different species (Taton et al., 2003). The ITS sequences show large variations in length and sequence between species, and they are easier to amplify because the rRNA operons are present in several copies. Based on ITS sequences and ARDRA fingerprinting analysis, 54 strains of the *Arthrospira* genus from four continents have been divided into five clusters grouped in two main genetic variants: IA, IB, IIA, IIB, IIA/IIB (Scheldeman et al., 1999; Baurain et al., 2002) and to which the new genetic variant III was later added (Comte et al., 2013). A study conducted by Manen and Falquet (2002) on the *cpcBA* locus reaffirmed that *Arthrospira* genus consists of three genetically clustered lineages. However, there was little overlap between the studied strains and the congruence between lineages observed on the basis of the different markers could not be assessed.

The usefulness of the *cpc*BA-IGS locus and ITS in *Arthrospira* phylogeny was presented by Dadheech et al. (2010). Thirty-three *Arthrospira* strains collected from North America, Africa and India were analyzed in comparison to 53 known *Arthrospira* strains using morphological traits and molecular phylogeny. Phylogenetic trees obtained in this study, one based on *cpc*BA-IGS and the second on the ITS region, were divided into two clusters—cluster I and cluster II, which is congruent with findings of Baurain et al. (2002) and Manen and Falquet (2002). Interestingly, the phylogenetic cluster division was related to *Arthospira* strains' origin. Strains in cluster I were mainly collected from Mexico, USA, or Peru, whereas in cluster II, they were collected from China, India, Kenya, or Chad, suggesting different evolutionary tracks of the two clusters.

### Transformation

The first attempt to build an *Arthrospira* DNA library was made by Kawata et al. (1998), with a method based on sonication, usage of TaqDNA polymerase, and ligation with a TA vector (Kawata et al., 1998). Further studies tested transformation methods based on electroporation. The method proposed by Kawata et al. suggested the use of a few elements (1) electroporation procedure, (2) elements of transposition system: natural Tn5 transposon and transposase, (3) cation liposome complexes, (4) and finally the *cat* reporter gene with chloramphenicol acetyltransferase (Toyomizu et al., 2001a; Kawata et al., 2004). However, this protocol was not reproduced. Recent study of Jeamton et al. (2017), resulted in an efficient and stable transformation of *Arthrospira platenis* C1. This protocol was also based on electroporation, but its novelty was the use of a type I restriction-modification systems inhibitor and liposomes to protect the DNA.

### DNA libraries, mutagenesis, and restriction-modification systems

Since 1981, various *Arthrospira* mutagenesis studies have been performed, mainly by Riccardi et al. NGT (nitrosoguanidine) was used as mutagenic agent, and resulted in *A. platensis* mutants resistant to: 5-fluorotryptophan, β-2-thienylalanine, ethionine, p-fluorophenylalanine, or azetidine-2-carboxylic acid (Riccardi et al., 1981, 1982). These mutants have been divided into two groups: mutants resistant to more than one analog, and mutants resistant to only one analog, both overproducing the corresponding amino acid. Researchers' attention focused on proline overproducing mutants, belonging to the second group. Some studies suggested that proline may be responsible for osmoregulation in blue-green algae (Gilles and Gilles-Baillien, 1985; Singh et al., 1996). Growth tests showed that, while control cultures' growth decreased with increasing NaCl concentration, proline-overproducing mutants' growth remained constant. Singh et al. (1996) revealed that a mutant strain of *Nostoc muscorum* with proline overaccumulation presented enhanced salinity tolerance. This suggests that simple genetic approaches, like point mutations, could be a solution for culture growth problems (Vonshak, 1997).

In 2008, via UV mutagenesis, Choi et al. obtained a mutant strain of *A. platensis* PCC 9108 that was able to grow mixotrophically in a SOT medium with glucose addition. The study revealed that the mutant biomass growth was significantly higher than a wild-type *Arthrospira*. Furthermore, the γ-linolenic acid (GLA) content (increasing with a higher dose of glucose in the medium) was two-fold higher in autotrophic culture and three-fold higher in mixotrophic one in comparison to the wild type. This suggests that the mutant strain possesses the ability to easier assimilate and metabolize glucose and presents an increased production of GLA (Choi et al., 2008).

Genetic studies have revealed the activity of restriction-modification (RM) systems in filamentous cyanobacteria (Zhao et al., 2006). Those bacterial systems protect cells from exogenous DNA. They consist of two enzymes: a restriction endonuclease, cutting the DNA at a specific site, and a methyltransferase, adding a methyl group to cytosine or adenine in the autochtonous DNA strand, preventing it from being cut. Restriction-modification systems are important in genetic studies, molecular cloning and are a perfect model for examining DNA-protein interactions (Wilson, 1991; Pingoud and Jeltsch, 2001; Waleron et al., 2006). In the study of Zhao et al. (2006), *Synechocystis, Synechococcus Prochlorococcus, Anabaena, Nostoc, Spirulina*, and *Trichodesmium* strains were examined *in silico*, using the RM gene set obtained from CyanoBase. The (BLAST)P and TBLASTN tools were used to obtain 30 putative RM genes, mostly type I RM systems. Additionally, gene expression profiles for “*Spirulina”* methyltransferases were characterized by the RT-PCR method (Zhao et al., 2006).

A recent study of Shiraishi and Tabuse (2013) revealed the presence of a new RM system in the *A. platensis* NIES-39 genome, named AplI. The NIES39_K04640 gene, a putative type II restriction enzyme, showed an endonuclease activity specific at the 5′-CTGCAG-3′ sequence.

### Genome studies

Hitherto seven *Arthrospira* genomes have been fully sequenced: *Arthrospira* sp. PCC8005, *A. platensis* NIES-39, *A. platensis* C1, *A. maxima* CS-328, *A. platensis* Paraca P0 (WGS), *A. platensis* YZ, and *Arthrospira* sp. TJSD091 (Fujisawa et al., 2010; Janssen et al., 2010; Carrieri et al., 2011; Cheevadhanarak et al., 2012; Lefort et al., 2014; Dong et al., 2015) (Table 1). According to the statistical comparisons (Cheevadhanarak et al., 2012), genome sizes of the *Arthrospira* strains range from 4.9 Mbp (*A. platensis* Paraca P0) to 6.8 Mbp (*A. platensis* NIES-39). The genome is organized as a single, circular chromosome and cells contain no known plasmid DNA. The total gene numbers vary from 5,401 to 6,676, and the percentage of protein-coding genes reaches up to 99% (Fujisawa et al., 2010; Janssen et al., 2010; Carrieri et al., 2011; Cheevadhanarak et al., 2012; Lefort et al., 2014; Dong et al., 2015).

**Table 1:** Summary of statistics of available on NCBI *Arthrospira* genomes data.

**Strain**	***Arthrospira maxima* CS-328**	***Arthrospira platensis* C1**	***Arthrospira platensis* NIES-39**	***Arthrospira platensis* str. Paraca**	***Arthrospira platensis* YZ**	***Arthrospira* sp. PCC8005**	***Arthrospira* sp. TJSD091**
Number of scaffolds	129	1	1	268	1	1	359
Number of contigs	129	63	18	208	10	6	296
Largest contig	324197	799912	870292	297520	2167980	2117149	164291
Total length	6003314	6089210	6788435	6501886	6520772	6228153	5978827
GC (%)	44.76	44.68	44.27	44.3	44.19	44.73	44.75
N50	92573	206210	619347	72660	1054592	1412831	50394
ITS cluster (after Baurain et al., 2002)	I.A	I.A	II.A	II.A	II.A	I.B	I.A
Sequencing method	n.a.	Sanger/454 GS FLX Titanium	ABI 3730	Illumina HiSeq	ABI 3730/Illumina GAIIx	n.a.	Illumina HiSeq
coverage	n.a.	28x	11x	36x	86x	n.a.	130x

All genomes contain highly interspersed repetitive sequences (reaching up to 9% of the genomes), including group II introns, phage-like sequences, insertion elements and, on average, six to 13 CRISPRs (Clustered Regularly Interspaced Short Palindromic Repeats). Together with RM systems, they compose a cellular defense system, which is a major barrier in the transformation of cyanobacterial cells (Fujisawa et al., 2010; Janssen et al., 2010; Cheevadhanarak et al., 2012; Lefort et al., 2014).

### Phages

Little is known about *Arthrospira* cyanophages. The first report about a virus infecting *A. platensis* was published by Jacquet and collaborators in 2013. The discovered phage was isolated from commercial culture ponds located in the South of France. On the basis of molecular and morphological characteristics, it was thought to be a member of the cyanopodovirus group, but finally, it appeared to be a very narrow host specific virus (Jacquet et al., 2013). Kaggwa et al. (2013) and Peduzzi et al. (2014) noticed morphological changes in *Arthrospira fusiformis* from African saline lakes connected with cyanophage infections. Infected cells formed long, narrow coiled filaments. Lysis and sudden breakdown in *A. fusiformis* biomass, which is a main food supply for flamingos, may cause a periodical decrease in their populations in African lakes (Peduzzi et al., 2014).

### Gas vesicles

In various aquatic prokaryotes, including *Arthrospira*, gas vacuoles are present and provide buoyancy to the cells. In the *Arthrospira* genus, gas vesicle clusters are dispersed throughout the cells, although their occurrence is dependent on culture conditions and may be lost as a result of mutations (Castenholz et al., 2001). Walsby (1994) revealed that there are two main gas vesicle proteins, GvpA, the small rib protein, and GvpC, the large outer surface protein. The most characteristic feature of GvpC protein is the presence of a highly conserved motif of 33 amino acid residues, forming tandem repeats. Miklaszewska et al. (2012) have proposed the following structure for the *gvp* operon: *gvpA1-gvpC1-gvpA2-gvpC2-gvpA3-gvpC3-gvpN*, which was identical in five analyzed *Arthrospira* strains. Although the operon could not be assembled during genome analysis, its structure could be determined by gene cloning. Additionally, the comparison of the *gvp* genes sequence between five *Arthrospira* strains (PCC 8005, PCC 7345, PCC 9108, PCC 9444, Moz 2.1) revealed the presence of polymorphic positions characteristic for different genetic clusters (I and II).

### Stress adaptation

Members of the *Arthrospira* genus are known from their resistance to different environmental conditions: high temperature, alkaline pH, high salt concentration. Genome studies revealed potential genes responsible for the adaptation capacity of *Arthrospira*. Nap-A type Na+/H+ antiporters of the membrane transporter family are known to be involved in salt tolerance at alkaline pH in several cyanobacterial species. Accumulation of bicarbonate in the cytoplasm additionally influences photosynthesis in high pH conditions. Homologs of these antiporters have been found in all *Arthrospira* genomes. *A. platensis* NIES-39 genome possesses seven genes encoding putative Na+/H+ antiporters, as well as two sets of genes for CO_2_ uptake (NDH-1) (Furrer et al., 2007; Fujisawa et al., 2010).

Another strategy for the tolerance to high salinity, in addition to ion transport, is the accumulation of compatible solutes in cyanobacterial cells. Compatible solutes are organic, highly water soluble compounds, that counterbalance the osmotic potential and maintain the cellular turgor. Those molecules also exhibit protective effects toward some sensitive macromolecules, what enhances their role in salinity stress tolerance. The genes responsible for the biosynthesis of three compatible solutes (trehalose, glucosylglycerol, glucosylglycerate) were detected by Hagemann, 2010, 2013) in four *Arthrospira* genomes (*Arthrospira maxima* CS-328, *Arthrospira sp*. PCC8005, *A. platensis* NIES-39, *A. platensis* Paraca P0). In *A. platensis* NIES-39 and *Arthrospira* sp. PCC8005, the trehalose biosynthesis pathway seems incomplete (Hagemann, 2010, 2013).

### Nitrogen fixing and hydrogen production

*Arthrospira* was originally described as a non-nitrogen fixing species, however, some genes responsible for heterocyst maturation (*patU, hetR, hetF*) and nitrogen fixation are conserved in all *Arthrospira* genomes. This fact suggests that heterocyst and trichome formation might be coupled (Fujisawa et al., 2010).

Analysis of *A. platensis* NIES-39 and *Arthrospira* sp. PCC8005 genomes revealed the presence of hydrogenase genes (*hox* and *hyp* loci). This suggests that *Arthrospira* is a potential organism for clean energy production (Fujisawa et al., 2010; Janssen et al., 2010). Indeed, *Arthrospira* strains are now being tested for hydrogen production (Juantorena et al., 2007; Raksajit et al., 2012).

Carrieri et al. (2011) have shown an increased autofermentation of the carbohydrates (glycogen and sugars) produced by the photosynthetic pathway under dark, anoxic conditions in *A. maxima* CS-328. By replacing potassium with sodium in cultivation buffers, these authors obtained high (67% higher than in normal conditions) yields of hydrogen, acetate, and ethanol. Removal and blocking of a sodium ion gradient resulted in increased catabolism of intracellular carbohydrates by *Arthrospira* cells. Additionally, genome sequencing studies revealed the presence of e.g., homologs of P-type ATPase that extrude sodium at the expense of ATP hydrolysis; genes homologous to *bicA* that encode sodium-bicarbonate symport protein; genes encoding a Mrp complex that play a role in extruding sodium under anoxic conditions (Carrieri et al., 2011).

### Toxin production

Genome analyses of *A. platensis* C1 and *A. platensis* NIES-39 also revealed the absence of genes responsible for the biosynthesis of toxins: non-ribosomal peptide toxins, polyketide toxins, urea-derived toxin and others, what makes the *Arthrospira* genus safe for biomass and food production. However, genomic studies showed the presence of genes encoding proteins containing an RTX-motif, that is characteristic for hemolysins (Fujisawa et al., 2010; Cheevadhanarak et al., 2012), though toxicity analysis proved that *Arthrospira* is safe to consume (FDA, 2002). RTX-motif proteins are also thought to play a role in plant nodulation and cyanobacteria motility, which may explain their presence in *Arthrospira* genomes (Hoiczyk and Baumeister, 1997; Linhartová et al., 2010).

The first cyanobactin cluster described in the cyanobacterial phylum was the patellamide group of bactins. Cyanobactin gene cluster consists of at least few elements: a precursor peptide gene and two short conserved hypothetical proteins named by similarity to the canonical patellamide cluster (Donia and Schmidt, 2011). In the *Arthrospira* genus, the *art* gene cluster (arthrospiramide) shows the features typical for cyanobactins, except for the presence of additional precursor peptides and a transposase gene between *artF* and *artG*. Although cyanobactins from *Arthrospira* genus encoded by the *art* gene cluster have been purified, their pharmacological properties should be further investigated (Donia and Schmidt, 2011). Based on a study of the N-terminal gene phylogeny, arthrospiramide was classified into cluster VI within already identified cyanobactins (Martins et al., 2013).

### Proteomics and transcriptomics

The first proteomic study of *Arthrospira* in light/dark diurnal cycles based on shotgun proteomics has recently been published. Examination concerns *Arthrospira* sp. PCC8005 strain and is based on fractionation workflows methods: gel-free and gel-based protein/peptide, combined with Liquid Chromatography-Tandem Mass Spectrometry (LC-MS/MS). Each of the methods provided a different number of identified proteins. Overall, the combined analysis resulted in the identification of 1,306 proteins. The researchers suggested that it represents a 21% coverage of the theoretical proteome of *A. platensis* PCC8005 (Matallana-Surget et al., 2014). Further study revealed that regulation of the 30 identified proteins differed during the light/dark growth transition. This effect was mainly observed for proteins related to Calvin cycle, photosynthesis, and translation processes. Genome studies indicated that *Arthrospira* contains the *kaiABC* genes, responsible for controlling the circadian clock. Interestingly, only one biological replicate showed the presence of *kai* proteins, whereas an inference of gene regulation was impossible. The study revealed that *kaiA* gene maintains a periodic expression under constant light conditions and that the overall *A. platensis* responses to LD (light/dark) cycles were correlated with the Earth conditions. The researchers suggest that further studies are needed to maintain a suitable and efficient bioreactor for long-term manned missions in space (Matallana-Surget et al., 2014).

Another proteomic study of *Arthrospira* was focused on protein expression under salt stress (Wang et al., 2013). The strain *A. platensis*-YZ was grown under salt-stress conditions: 0.02, 0.5, 1.0 M NaCl, and the protein expression patterns were compared. The study revealed that 114 proteins out of 141 were homologous to the ones in other *Arthrospira* strains (*A. platensis* Paraca and *A. maxima* CS-328), but the others were thought to originate from the other bacteria. The expression level of 29 genes responsible for salt-stress response was measured by qRT-PCR. The results showed that the transcription level of 11 genes remained up-regulated, and expressions of all 12 examined genes were consistent at their transcription and protein level. Proteins studied by Wang et al. (2013) were classified into 18 types according to their function (such as carbohydrate transport and metabolism, cell envelope biogenesis, post-translational modification or translation, ribosomal structure), and they were involved in 31 different metabolic pathways.

Huili et al. (2013) studied by proteomics the temperature response at 15, 35, and 45°C of *A. platenis* YZ strains. The expression of 122 proteins was significantly affected at different temperatures. In this study, the majority of the genes were homologous to those from other *Arthrospira* genomes (116 homologous genes out of 122 examined). Proteins showing different expression with changing temperatures appeared to be involved in different functions. This included the post-translational modification (e.g., chaperones), translation (e.g., ribosomal biogenesis), carbohydrate transport, energy metabolism (e.g., respiratory electron transport), and others that were classified in 14 different types depending on the functions by the COG database. Likewise, in the previous study of salt-stress (Wang et al., 2013), the transcription and translation levels of 26 proteins measured by qRT-PCR remained constant.

Transcriptome in different stress conditions have been studied e.g., N-limited stress (Deschoenmaeker et al., 2014; Depraetere et al., 2015), high temperature (Panyakampol et al., 2014), and sulfur stress (Kumerasan et al., 2017). Down-regulation of *de novo* protein synthesis under N-limited stress has been revealed by Depraetere et al. (2015). Study of Panyakampol et al. (2014) suggest up-regulation of thermal-responsive genes helps the cells to remain in homeostasis during high-temperature conditions. Group of Kumerasan et al. (2017) observed that sulfur stress decreases level of pigments and reduces the biomass growth. Additionally, they revealed that *Arthrospira* was able to survive the stress due to expression changes of genes related with sulfur metabolism.

Majority of the mentioned transcriptomic studies were based on microarray assay, so it is relevant to mention that complete transcriptome of *Arthrospira* genus remains unavailable.

Due to the mass-scale character of commercial *Arthrospira* cultivation, understanding of the mechanisms that underlie responses to environmental changes is essential. In response to that, group of Senachak et al. (2015) developed an online platform, SpirPro, with integrated proteome and PPI database of *A. platensis* C1, publicly available on the web.

## Cultivation methods

### Light intensity

Light, temperature, and nutrient availability are considered to be major limiting factors for *Arthrospira* growth in adequate culture media. *Arthrospira*, a photosynthetic organism, needs light as the main energy source and many studies were focused on light intensity effect on the increase in biomass production. In laboratory conditions, however, some discrepancies were observed concerning the most favorable light intensity for optimal biomass production. Zarrouk in his doctoral thesis determined an optimal photosynthetic photon flux (PPF) at 480–550 μmol m^−2^ s–^1^. More recent studies report an optimal light intensity for maximal biomass production that varies between 37 μmol m^−2^ s^−1^ (Kumar et al., 2011) and 150–200 μmol m^−2^ s^−1^ (Markou et al., 2012). Those values correspond to the light intensity of an overcast day and of a sunny day, respectively. These differences in reported values may be a result of using different sources of light and different strains of *Arthrospira*. However, at some point, photoinhibition, loss of photosynthetic capacity due to damage caused by photon flux, can be observed. Consequently, reduction of biomass production is observed (Vonshak, 1997). *Arthrospira* cultures grown in outdoor ponds at higher cell density providing self-shading or/and with net shading showed a lower reduction in the Fv/Fm ratio (parameter describing the maximal photochemical efficiency of PSII), that was helpful in maintaining suitable biomass productivity (Vonshak et al., 2014). This approach allowed to obtain a higher biomass yield by comparison to indoor production. Additionally, light intensity has been proven to have a linear effect on extracellular polymeric substances (EPS) production by *Arthrospira*. According to Trabelsi et al. (2009), a light intensity of 180 μmol m^−2^ s^−1^ and presumably higher values result in a significant increase of EPS production.

### Led light of different colors

Although fluorescent lamps seem to be the most popular light source for *Arthrospira* cultivation in laboratories, it can also be grown under illumination provided by different light sources like e.g., light emitting diode (LED). In a study, the effect of a mono-, di-, and polychromatic LED light (of average intensity 166 μmol m^−2^ s^−1^) on productivity and cell composition was determined. The greatest increase in biomass production was observed in cultures illuminated with red and pink light. Cultures illuminated with yellow and white LED light showed almost a two times lower productivity. The lowest increase in biomass production was observed under blue LED light. This difference in growth rate is probably a result of pink and red LED emitting in close proximity to chlorophylls (435/676 nm) and phycocyanin (620 nm) absorption peaks, while other colors emit at wavelengths relatively distant (Markou, 2014). Chen et al. (2010) and Wang et al. (2007) obtained similar results, while Ravelonandro et al. (2008) and Madhyastha and Vatsala (2007) described best biomass productivity under cultivation with green and blue light illumination. This contradiction probably is a result of using different light sources. It is also worth pointing that in many reports, the light sources' descriptions are neglected as only the irradiance level is provided, which may lead to bad reproducibility of the obtained results.

Changes in light color used for culture illumination also have an impact on cell composition. Cultures of *A. platensis* cultivated under different light colors showed no changes in carotenoid composition. However, blue LED illumination resulted in the highest content of, not only chlorophyll and phycocyanin, but also carbohydrates and lipids. That can be explained as an attempt by the cyanobacteria to improve the efficiency of photosynthesis in less than optimal light conditions. The highest protein content was obtained under green and white LED illumination (Markou, 2014).

### Effect of the light intensity on biomass growth under mixotrophic conditions

It is already known that *Arthrospira* can grow in three modes: autotrophic—based on photosynthesis, heterotrophic—depending on organic carbon sources (Vonshak et al., 2000; Chojnacka and Noworyta, 2004; Andrade and Costa, 2007), and mixotrophic, which use those two modes simultaneously (Marquez et al., 1993). Mixotrophic cultivation of *A. platensis* results in higher overall biomass production in any light intensity used compared to autotrophic and heterotrophic cultures (4 klux for (Chen, 1996); below 150 μmol m^−2^ s^−1^ for (Vonshak et al., 2000)). Indeed, the maximal biomass yield was 2.3 times greater in mixotrophic conditions under 150 μmol m^−2^ s^−1^ light intensity (Rym et al., 2010). The results showed that the amount of biomass obtained under mixotrophic conditions was a result of the combined response of cyanobacterium to light and organic carbon source—glucose. Moreover, the addition of glucose to the cultivation medium in mixotrophic conditions under a light intensity of 150 μmol m^−2^ s^−1^ resulted in higher EPS yield compared to autotrophic and heterotrophic conditions (Trabelsi et al., 2013). Mixotrophic cultures also have lower light requirements, higher oversaturation levels and can recover quicker from light stresses than autotrophic cultures (Vonshak et al., 2000). Under lower illumination, glucose has a low influence on the maximal biomass concentration and growth rate (Rym et al., 2010).

### Effect of UV radiation on morphology

Ultraviolet light is a naturally present environmental factor influencing the outdoor growth of *Arthrospira*. Cyanobacteria developed various defense mechanisms to protect themselves. First, the cells may avoid the stress-inducing or damaging factor by changing the buoyancy of the trichome or its rotation (Miklaszewska et al., 2012). Secondly, cells may repair the damaged DNA or synthesize DNA *de novo* (Sinha and Häder, 2002). Another defense mechanism is the production of UV-absorbing compounds like mycosporine-like amino acids (MMAs), scytonemin (Rastogi and Incharoensakdi, 2014) and carotenoids in higher quantity (Rakhimberdieva et al., 2004; Gao and Ma, 2008).

Regardless of its impact at the molecular level, UV is causing morphological changes in the cells that are visible after 2 h of exposure. The exposure to full solar radiation (UV-B, UV-A, and PAR) has the most destructive effect and leads to severe filaments breakage. In contrast, longer exposure of filaments to only PAR only leads to slight breakage (Wu et al., 2005). In addition, Sarma et al. (2010) showed that, to some extent, PAR light protects filaments from breakage caused by UV radiation when applied simultaneously. It is important to emphasize that the destructive effect of UV is more pronounced at 15–23°C—temperatures below *Arthrospira* growth optimum. In temperatures around its optimum, the damages caused by UV exposure are smaller and become visible only after a longer exposure—up to 8 h (Gao et al., 2008). UV light exposure results in the formation of a tighter helical structure of trichome (Wu et al., 2005; Helbling et al., 2006) and it has already been suggested that this change in morphology is increasing the tolerance to high-light conditions (Jeeji Bai and Seshadri, 1980). Cultivation in the laboratory without UV radiation seems to promote the development of straight filaments (Lewin, 1980; Jeeji Bai, 1985).

### Growth in different salinities

*Arthrospira* is known as an alkalophilic and halophilic organism. *Arthrospira* species have been reported to grow in the natural environment in a salinity range from 1.1 to 300 g/L, understood as a total amount of all salts present in the environment (Dadheech et al., 2010). In laboratory conditions, according to literature data, *Arthrospira* is grown in media with salinity levels varying from 8.5 to 200 g/L (Arruda et al., 2009; Pelizer and Moraes, 2009). Many studies focused on establishing the optimal salinity to obtain the highest *Arthrospira* biomass yields and different media with and without supplementation were utilized. Among the most commonly used media are the ones by Zarrouk, Schlösser, Provasoli, Paoletti, and Spirulina Medium (Zarrouk, 1966; Sánchez-Luna et al., 2004; Converti et al., 2006; Tambiev et al., 2011; Ogato et al., 2014). The composition of those media only slightly varies and all of them contain a substantial amount of salts. It is difficult to define which medium is the best since results are contradictory and seem to differ not only between media but also between strains. The biomass yield obtained is most likely dependent upon multiple factors like light intensity, nitrogen, and micronutrients availability, stirring, and pH (Converti et al., 2006; Ravelonandro et al., 2011; Moraes et al., 2013).

Cultivation of *Arthrospira* in synthetic media is expensive and not economical. Therefore, numerous studies were designed to find the best natural alternative medium without compromising biomass productivity (Materassi et al., 1984; Tredici et al., 1986; Costa et al., 2003; Ogato et al., 2014). A first choice alternative medium would be lake or seawater with proper chemical composition. An example of such studies is the cultivation of *Arthrospira* in waters from two saline-alkaline lakes located in the Ethiopian rift: Chitu, in which a natural population of *A.fusiformis* is present and the nearby Shalla, which was devoid of *Arthrospira*. After supplementing the water from these lakes with a synthetic medium, the yield was comparable to that obtained from the control grown in standard Spirulina medium (Ogato et al., 2014).

### Nitrogen source

Strains of the genus *Arthrospira* are usually cultivated in media containing KNO_3_ or NaNO_3_ as a primary nitrogen source. However, reports concerning *Arthrospira* growth in media with different and cheaper nitrogen source are fairly abundant. An extensively studied nitrogen source is urea (Feng and Wu, 2006; Sánchez-Luna et al., 2007; Avila-Leon et al., 2012) as it is quite cheap. However, at higher concentrations, it tends to become toxic because it damages the Photosystem II (Dai et al., 2014) and under alkaline conditions, urea hydrolyses to ammonia, which results in a loss by off-gassing (Danesi et al., 2002). Loss of ammonia to the atmosphere is also a problematic issue if ammonium salts are used. Markou et al. (2014) showed that the ammonium chloride loss as gaseous ammonia ranged from about 17 up to 80%, depending on the initial salt concentration. In another study, Bezerra et al. (2008) also used ammonium chloride as a nitrogen source, but have not reported loss by off-gassing under non-light limited conditions during the fed-batch experiment. Cultivation of *Arthrospira* in waters from the Gulf of Mexico supplemented with anaerobic effluents after pig waste digestion under two light intensities (66 and 144 μmol m^−2^ s^−1^) resulted in significant increases in lipids and polysaccharides (Olguín et al., 2001) as compared to the biomass grown in control conditions. Another study investigated the growth of *Arthrospira* in nitrogen—depleted medium and the effect of such cultivation on cells (Deschoenmaeker et al., 2014). The strain PCC8005 was able to survive long—term cultivation without any external nitrogen source and recovered upon transfer to standard medium. Nevertheless, the lack of nitrogen source resulted in a decrease of protein and phycobilisome contents, downregulation of the inorganic carbon fixation pathway and glycogenolysis as well as upregulation of glutamine synthesis, nitrogen metabolism, carbohydrate synthesis, and both HglK and HglR proteins, which are involved in heterocyst formation in other cyanobacteria.

### Indoor and outdoor cultivation methods

Mass cultivation of photosynthetic organisms such as *Arthrospira* has been widely studied in order to establish the most economical process to obtain biomass. For indoor cultivation, closed photobioreactors are the most popular choice. They allow the control of cultivation conditions such as pH, light intensity, or CO_2_ supplementation as well as contamination prevention (Travieso et al., 2001). However, cultivation at the industrial scale is most often performed in large outdoor open-ponds (Borowitzka, 1999). Due to the fact that *Arthrospira* requires high temperatures in order to grow efficiently, it is often cultivated in regions with high average air temperature and high amounts of sunlight (tropical and sub-tropical regions), which lowers the production costs significantly. However, though economically more sustainable, open-pond cultivations also face certain problems. Those issues, like contaminations, mixing of the cultures, CO2 utilization efficiency and weather influence have been discussed in a review about engineering solutions for open microalgae mass cultivation (Apel and Weuster-Botz, 2015). Engineerig solutions for the above-mentioned problems were presented and a model-based bioprocess and bioreactors development was proposed as a promising tool for further improvement in productivity of microalgae during mass cultivation.

Industrial production of “Spirulina” has existed for many years in countries such as Australia, India, Israel, Japan, Malaysia, China, Chad, and Myanmar, without production data being reported to FAO. The first pilot plant started production in 1973 in Mexico and produced 150 tons of dry “Spirulina” biomass per year. The production increased later to 300 tons per year (Habib et al., 2008). In 1988, yearly worldwide production was estimated on 900 tons of dry biomass (Vonshak and Richmond, 1988; Pulz and Gross, 2004). Between 2000 and 2004 the world production raised to 3,000 tons per year (Pulz and Gross, 2004; Shimamatsu, 2004). In 2010 efficiency of dry mass production reached 90 000 tons and remained at this level until last reports from FAO (2016). The largest farm is estimated to produce 500 tons of dry biomass per year (FAO, 2016).

## *Arthrospira*, human health, and medicine—the multiplot story

Because of its chemical compound richness, the members of the *Arthrospira* genus are potent candidates as sources for bioactive substances. In 1993, the World Health Organization^1^ announced that *Arthrospira* (formerly named *Spirulina*) is an “interesting food for multiple reasons, rich in iron and protein, and is able to be administered to children without any risk” (WHO; Credentials|Abundance, 2017). Since then, *Arthrospira* products are treated as a superfood—a nutrient-rich food considered to be especially beneficial for health and well-being' (definition of superfood by Oxford Dictionary^2^). The American Food and Drug Administration classified *Arthrospira* preparations as GRAS (Generally Recognized As Safe) in 2002 (FDA). In 2011, a commission of United States Pharmacopeia reviewed information from human clinical trials, animal studies, and regulatory and pharmacopeial sources and analyzed 31 adverse event reports regarding “Spirulina” to assess potential health concerns. As a result of the performed investigation, the commission assigned a Class A safety rating for *S. maxima* and *S. platensis*, thereby permitting the admission of quality monographs for these dietary supplement ingredients in USP-NF (United States Pharmacopeia and the National Formulary) (Marles et al., 2011).

The pharmacological potential of pure *Arthrospira* biomass, hot water extracts, purified polysaccharides, phycocyanin, and calcium spirulan preparations has been examined in many studies. Results reveal the ability of these preparations to beat viral infections, act as immunomodulatory factor, antitumor agent, genetic cell damage and cardiovascular diseases protector. *Arthrospira* powder sold as “Spirulina” in general has no detectable adverse effects on human body, reproductive performance, embryo/fetus development, and growth. Nevertheless, in cultures without GMP (Good Manufacturing Practice) application, *Arthrospira* biomass may contain low levels of mercury and other heavy metals that constitute a direct health danger (Kim and Lee, 2013; Vicat et al., 2014). Nonetheless, *Arthrospira* may become an important source of food and feed in the future, as 4 m^2^ basin produces around 40 g of dry biomass daily with 10 times lesser requirements of water in comparison to equivalent weight of soybean (Habib et al., 2008).

In the presented review, we aim at demonstrating the broad medical potential of *Arthrospira* biomass, extracts, and products, summarized in Table 2. Due to the diversity of material used in research—from pure laboratory strains to commercial products sold as “Spirulina,” these authors decided to reproduce the nomenclature of the cited original papers.

**Table 2:** Therapeutic effects of the most important *Arthrospira* components: immulina, calcium spirulan C-phycocyjanin, and gamma linoleic acid as well as extracts and supplementary formulations.

	**Immunomodulatry effect**	**Antimicrobial activity**	**Anticancer effect**	**Cardiovascular system protection**
Calcium spirulan		Selective inhibition of the penetration and replication of enveloped viruses such as Herpes simplex virus type 1 (HSV-1) and type 2 (HSV-2), Human cytomegalovirus (HCMV), measles, mumps, influenza A, and HIV-1 (Hayashi et al., 1996; Hernández-Corona et al., 2002; Rechter et al., 2006).	Inhibition of the invasion of B16-BL6 melanoma, colon carcinoma, and HT-1080 fibrosarcoma cells (Mishima et al., 1998).	
Immulina	100- to 1,000-fold more potent monocyte activator *in vitro* than standard polysaccharide preparations in clinical cancer immunotherapy (Løbner et al., 2008). Increase of TNFα, IFNγ, and IL-6 levels in blood (Løbner et al., 2008).	Strong *in vitro* activity against *Candida albicans* and the tetanus toxoid (Løbner et al., 2008).	*Look at the immunomodulatory effect*.	
C-phycocyanin	Stimulation of the production of the pro-inflammatory cytokines (Chen H. W. et al., 2014). Macrophage, T, and B cells activation (Khan Z. et al., 2005). Reduction of the allergic inflammations by suppressing the antigen-specific IgE antibodies (Nemoto-Kawamura et al., 2004). Histamine release inhibition from mast cells (Nemoto-Kawamura et al., 2004). Reduction of functional neutrophils metabolic activity (Dartsch, 2008).	Inhibition of bacterial growth in many drug-resistant strains such as *E. coli, K. pneumoniae, P. aeruginosa*, and *S. aureus* (Sarada et al., 2011).	Cell cycle arrest and mediation of apoptosis (Ouhtit et al., 2014; Pan et al., 2015; Saini and Sanyal, 2015). Antiproliferative effect in different cancer cell lines (Subhashini et al., 2004; Li et al., 2006, 2013; Roy et al., 2007; Chen and Wong, 2008; Saini and Sanyal, 2014; Yang et al., 2014; Pan et al., 2015). Anti-angiogenic role in cancer model by inhibiting cyclooxygenase-2 (Saini and Sanyal, 2014). Antineoplastic effects via the regulation of membrane properties (Saini and Sanyal, 2014). Mimics cell intracellular bilirubin's role as an inhibitor of NADPH oxidase activity, what makes it a promising agent to mitigate the pro-oxidative effects of smoke aldehydes and ketones responsible for lung cancer development (McCarty et al., 2015).	Lowering of the cholesterol solubility and intake by Caco-2 intestinal cells (Nagaoka et al., 2005). Inhibition of the development of atherosclerosis (Li et al., 2013).
GLA			Potent cytotoxic agent against human lung carcinoma—A549 cell line (Jubie et al., 2015).	Reduction of the cholesterol accumulation in the hypolipidemic nephrotic syndrome (Samuels et al., 2002).
Other compounds	Polisachcaride increased the levels of IL-1, IL-3, and TNFα.	Phycobiliproteins isolated from *A. fusiformis* exhibited antibacterial activity against *Streptococcus pyogenes* (Najdenski et al., 2013).	Tetrapyrrolic compounds act against pancreatic cancer cells (Konícková et al., 2014).	
Supplements	Immulina®, a commercial extract of *Arthrospira (Spirulina) platensis* is a potent activator of THP-1 monocytes and CD4+ T cells *in vitro* and enhances several immunological functions in mice (Nielsen et al., 2010).	May help patients with chronic HCV infection, but still further research is needed (Yakoot and Salem, 2012). Dry biomass exhibits antiviral activity on the bacteriophage T4 (Gorobets et al., 2002).		In patients with ischemic heart disease “spirulina” improves patients' lipid profiles and helps to lower blood pressure (Torres-Duran et al., 2007). Protective effect on doxorubicin-related cardiac side effects in rodents (Khan M. et al., 2005; Khan et al., 2006) as well as against heart attacks in humans (McCarty, 2010).
Extracts		Water, propanol and acetone extracts of *Arthrospira* showed antimicrobial activity against *Klebsiella pneumoniae, Proteus vulgaris, Pseudomonas aeruginosa, Salmonella* Typhi, *Staphylococcus aureus*, and *Escherichia coli*. (Mala et al., 2009). Butanol extract from “Spirulina” powder acts as an antifungal agent against *Candida glabrata* (Santhanam, 2011).	Potent anticancer activity in human colon adenocarcinoma (HT29) and human kidney adenocarcinoma (A498) cancer cell lines with no significant impact on healthy cells (Srivastava et al., 2015)	Water extracts lower the fat absorption by inhibiting pancreatic lipase activity (Han et al., 2006).

### Substances that play a pivotal role in *Arthrospira* multi-directional activity

The first analysis performed by Leonard and Compere (1967) showed that 45% of the dry biomass of *Arthrospira* was composed of proteins. Recent research revealed that the protein content may in fact be higher, up to 77% of the dry weight, depending on external conditions. Indeed, the analyzed protein content in all strains cultivated in the laboratory was higher than for the biomass collected from open ponds (Ciferri, 1983). The pigments content appear to depend on the temperature and light intensity (Kumar et al., 2011) and the amino acids content depends from the salinity (Volkmann et al., 2008; Wang et al., 2013).

The nutritional value of *Arthrospira* was reviewed by Miklaszewska et al. (2008a). Here we present the most important dietary aspects. *Arthrospira* is a valuable source of protein, not only because its high concentration in the dry biomass but also thanks to the amino acids composition and high digestibility. It is worth mentioning that *Arthrospira* biomass contains all 8 exogenic and 12 endogenic amino acids. The most abundant amino acids are leucine (10.9%), valine (7.5%), and isoleucine (6.8%). Although the content of cysteine and methionine is the lowest of all present amino acids, it is still higher compared to cereals or vegetables.

The lipid content of *Arthrospira* biomass is difficult to determine due to the differences in efficiency of extraction methods and changes in its actual content. Reported data vary from 1.5 to 12% of dry mass (Ciferri, 1983). Notably, the gamma linolenic acid (GLA) synthesized by the delta-6 fatty acid desaturase can reach up to 40% of all fatty acids in *Arthrospira platensis* (Murata et al., 1996). It acts as a precursor of prostaglandins, tromboxanes, and leukotrienes. That makes *Arthrospira* one of the best-known sources of γ-linolenic acid. Additionally, *Arthrospira* extracts contain vitamin F, linolenic, and arachidonic acids, fatty acids that promote cholesterol normalization (Miklaszewska et al., 2008a). However, the presence of arachidonic acid may be the effect of improper sample treatment during the analytical procedure as cyanobacteria do not possess fatty acid elongases required for C20 fatty acids production (Iliev et al., 2011).

*Arthrospira* carbohydrates content vary from 10 to 15% of dry biomass, mainly as rhamnose and glycogen. An important carbohydrate, mesoinositol phosphate, reaches a concentration of 350–850 mg/kg dry biomass. This compound is a perfect source of organic inositol (Falquet and Hurni, 1997). High molecular polysaccharides extracted from the *Arthrospira* biomass or culture medium have anticancer, immunostimulatory effects and can constitute scaffolds for tissue/organ regeneration in regenerative medicine (de Morais et al., 2010, 2014; Nielsen et al., 2010; Kurd and Samavati, 2015).

Although proteins, carbohydrates, and lipids are the building material of the cell, other trace elements significantly impact human metabolism. *Arthrospira* contains many essential minerals and trace elements like potassium, zinc, calcium, magnesium, selenium, manganese, iron, phosphorus, that are absorbed from its culture medium and transformed into chelated, easily absorbed forms. Potassium is an essential enzyme activator in muscle and heart, responsible also for maintaining the electrolyte balance. Iron consumed with *Arthrospira* is two times better assimilated than the one eaten with vegetables or meat (Puyfoulhoux et al., 2001).

*Arthrospira* cells contain carotenoids in different forms: α-carotene, β-carotene, cryptoxanthin, zeaxanthin, xanthophylls, echinenone, lutein. Eighty percent of all carotenoids present in *Arthrospira* are made by β-carotene (700–1,700 mg/kg of biomass) and cryptoxanthin (100 mg/kg), which are both precursors of vitamin A. *Arthrospira* supplements are efficient in eliminating vitamin A deficiency (Falquet and Hurni, 1997; Miklaszewska et al., 2008a; Naturalways, 2017).

*Arthrospira* biomass contains also other enzymatic pigments such as chlorophyll, phycocyanin, porphyrin. Chlorophyll supports intestine peristalsis, normalizes secretion of digestive acids, decreases secretion of pepsin and soothes inflammation. Porphyrin forms a nucleus of hemoglobin, what makes it an important factor in the maintenance of healthy red blood cells. Phycocyanin is related to bilirubin, essential for liver functions and digestion of amino acids. Apart from standard functions carotenoids and phycocyanin are antioxidants, reduce free radicals and have potential anti-tumor properties (Pervushkin et al., 2001; Miklaszewska et al., 2008a).

Besides its nutritional values, the consumption of *Arthrospira* as a food supplement gives other advantages. The cell wall of *Arthrospira* does not contain cellulose, which make it very easy to digest. Additionally, *Arthrospira* biomass has a very low level of nucleic acids and due to alkaline growth conditions, it is not easily contaminated by most of the human pathogens (Ciferri, 1983). However, in a recent report of the Vardaka research group, 31 dietary supplements sold as “Spirulina” on the Greek market contained 469 different bacterial OTUs. Among them were toxic cyanobacteria from the *Microcystis, Nostoc*, and *Anabaenopsis* genera. Moreover, supplements were contaminated with human pathogens (Vardaka et al., 2016). This highlights the need to control the quality of the production, as for any other foodstuff.

Detailed research has been conducted to confirm the therapeutic effects of some compounds that are unique for *Arthrospira*, including (I) calcium spirulan (II) immulina (III) C-phycocyanin and (IV) γ-linolenic acid (GLA).

Calcium spirulan (Ca-SP) is a complex polysaccharide composed of rhamnose, ribose, mannose, fructose, galactose, xylose, glucose, glucuronic acid, galacturonic acid, sulfate, and calcium (Lee et al., 1998, 2000). It was shown to inhibit the replication of enveloped viruses from different genera (Hayashi et al., 1996). Immulina is also a high-molecular-weight polysaccharide and exhibits significant effects as an immunostimulator (Nielsen et al., 2010). C-Phycocyanin is an unusual nontoxic fluorescent protein having an antioxidative, anti-inflammatory and antitumor potential (de Jesus Raposo et al., 2013). Studies suggest it is more potent in colon cancer prevention than vitamin E and germanium-132 (Chen and Zhang, 1995). The γ-linolenic acid controls cholesterol levels and protects the cardiovascular system (Białek and Rutkowska, 2015).

### *Arthrospira* empowers natural immunological responses

*Arthrospira* components are potent immunostimulating agents, they enhance the resistance to infections, have the capacity to influence hematopoiesis and stimulate the production of antibodies, especially IgA (Nemoto-Kawamura et al., 2004) and cytokines. Compounds from cyanobacteria are known to empower the immune system to inhibit carcinogenesis and assist natural healing mechanisms. Indeed, *Arthrospira* appears much more effective in Th1-response stimulation than Th2 (Mao et al., 2000).

Immulina is 100-1000x more active as monocyte activation factor *in vitro* than polysaccharide preparations that were being used at the time in clinical settings for cancer immunotherapy (Løbner et al., 2008). Immulina raises TNFα, IFNγ, and IL-6 blood levels (Løbner et al., 2008). Polysaccharide extracts from “Spirulina” significantly increased the levels of IL-1, IL-3, and TNFα.

The C-phycocyanin also exhibits potent immunostimulatory effects by stimulating the generation of pro-inflammatory cytokines, which in turn boost the cytotoxic functions of CTL (cytolytic T lymphocytes) and NK (natural killer) cells activity (Chen H. W. et al., 2014). Moreover, pro-inflammatory cytikines activate macrophages, T, and B cells (Khan Z. et al., 2005). Studies suggest that this multi-directional effect of *Arthrospira* consumption is due to an involvement in signaling responses through toll-like receptors in blood cells (Balachandran et al., 2006; Kawanishi et al., 2013).

*Arthrospira* extracts not only increase resistance to infectious diseases or natural healing mechanisms but also can modulate allergic responses. They sustain the functions of mucosal immunological mechanisms and reduce allergic inflammations by suppressing the antigen-specific IgE antibodies (Nemoto-Kawamura et al., 2004). So far, Mao et al. (2005) observed positive results with “Spirulina” dietary supplementation (product delivered by Earthrise Nutritionals, Inc., Irvine, CA) to protect against allergic rhinitis. Another anti-inflammatory effect of “Spirulina” phycocyanin is the histamine release inhibition from mast cells (Nemoto-Kawamura et al., 2004) and the reduction of functional neutrophils metabolic activity (four “Spirulina” preparations, Dartsch, 2008).

### Antiviral activity

Although its exact mode of action is unknown, scientists suspect that unique nutrients play an important role in *A. platensis* function as an antiviral (Blinkova et al., 2001). The calcium spirulan, isolated from hot water extracts is very effective against a variety of viruses and it inhibits the replication of enveloped viruses such as Herpes simplex virus type 1 (HSV-1) and type 2 (HSV-2), Human cytomegalovirus (HCMV), measles, mumps, influenza A, and HIV-1 in *in vitro* conditions (Hernández-Corona et al., 2002; Rechter et al., 2006). Study performed by Hayashi et al. (1996) indicated that calcium spirulan selectively inhibits the penetration of the virus particle into the host cell and is more effective than dextran sulfate. The most probable mode of action is the conformational chelation of sulfate groups on the surface of the virus envelope.

Encouraging results were observed when *Arthrospira* supplements were administered to patients with chronic HCV infection (Yakoot and Salem, 2012). Nevertheless, further research is needed to confirm this effect.

Interestingly, dried *A. platensis* exhibits antiviral activity on the bacteriophage T4 (Gorobets et al., 2002).

### Antibacterial and antifungal properties

Compounds isolated from *Arthrospira* cells can also act as antimicrobial agents. Animal studies suggest that they enhance natural immunological clearance mechanisms after bacterial infections with *Escherichia coli* or *Staphylococcus aureus* (Quereshi et al., 1995).

Mala et al. (2009) studied the antimicrobial activity of various organic and aqueous extracts of *A. platensis*. In agar-solid diffusion tests, a water extract of *Arthrospira* (WEA) showed a maximal antimicrobial activity (18.00 mm inhibition zone) in case of *Klebsiella pneumoniae* and a minimum activity against *Proteus vulgaris* (10.0 mm). A propanol extract (PEA) exhibited at least a 7.0 mm inhibition zone for *Pseudomonas aeruginosa* and 8.0 mm for *E. coli*. The acetone extract (AEA) displayed the highest biological activity with 17 mm inhibition zone against *K. pneumonia*, moderate activity in case of *Salmonella* Typhi and a 10.0 mm inhibition zone with *P. aeruginosa, E. coli*, and *S. aureus*. Sequential extracts of *A. platensis* exhibited the maximal antimicrobial activity and an inhibition zone of 25.3 mm was observed for *K. pneumoniae* and 16.0 mm for *P. vulgaris*. The authors assigned the antimicrobial activity to the peptides detected in the FTIR spectrum of this extract.

Microalgal cultures of *A. platensis* displayed significant antimicrobial activity against six *Vibrio* strains (Pradhan et al., 2011). Phycobiliproteins isolated from *A. fusiformis* exhibited antibacterial activity against *Streptococcus pyogenes* (Najdenski et al., 2013). The C-phycocyanin purified from *A. platensis* was able to inhibit the bacterial growth in many drug-resistant strains such as *E. coli, K. pneumoniae, P. aeruginosa*, and *S. aureus* (Sarada et al., 2011). Besides antimicrobial and antiviral properties, *Arthrospira* formulations show antifungal characteristics. Butanol extract from “Spirulina” powder was tested as a potential antifungal agent against *Candida glabrata* and caused a 13 mm growth inhibition zone in test plates (Santhanam, 2011). The previously mentioned immulina was found to be strongly active *in vitro* against *Candida albicans* and the tetanus toxoid (Løbner et al., 2008).

Recently, Duda-Chodak (2013) demonstrated that the water extract of *Arthrospira* has a notable impact on microorganisms. An inhibitory activity was shown against *Bacillus subtilis, Micrococcus luteus, Rhodotorula*, and *Penicillum*. Water extract of *Arthrospira* also strongly stimulated *Alicyclobacillus acidoterrestris*, and *Geotrichum* growth. Higher concentrations promoted the development of mycelium and production of conidiophores by *Cladosporium* and *Aspergillus niger*.

*A. platensis* was documented to be used as a bioreactor for production of silver nanoparticles. The latter are powerful toxic agents against the cancer promoter cyanobacterium, *Microcystis aeruginosa* (El-Sheekh and El-Kassas, 2014).

### Activity against tumors

Strong immunological influence and antioxidant activity predestinates *Arthrospira* to become a useful support for antitumor immunotherapy, both as a preventive and therapeutical agent. Konícková et al. (2014) demonstrated an antioxidative activity of *A. platensis* and its tetrapyrrolic compounds against pancreatic cancer cells. Kurd and Samavati (2015) showed that polysaccharides extracted from *A. platensis* had strong scavenging activities *in vitro* on DPPH (stable nitrogen-centered free radical) and hydroxyl radicals (but in this case lower than vitamin C). Studies indicate that *Arthrospira* or its components also have radioprotectant and cytotoxic properties. MB-6 is a novel herbal preparation containing fermented soybean extract, green tea extract, *Antrodia camphorata* mycelia, spirulina, grape seed extract, and curcumin extract. This supplement was proven to be a promising support that increases the effectiveness of chemotherapy in patients with metastatic colorectal cancer (Chen W. T. et al., 2014). Radioprotectant and cytotoxic effect of *Arthrospira* was observed in combined therapy with metronidazole in relapsed vulvar cancer (VVC) (Kiziltan et al., 2015).

Srivastava et al. (2015) screened five cyanobacterial strains, including *Arthrospira* sp. CCC729 for anticancer potential. Tests were performed on human colon adenocarcinoma (HT29) and human kidney adenocarcinoma (A498) cancer cell lines. Crude extracts and TLC eluates of *Arthrospira* sp. CCC729 exhibited potent anticancer activity. Apoptotic studies in cancer cell line (A498) and normal human epithelial cells (MCF-10A) revealed no significant impact on MCF-10A cells, in contrast to cancer cells.

During investigation performed on DMH-induced rat colon cancerogenesis, the C–phycocyanin promoted the arrest of the cell cycle by downregulating cyclin D1, cyclin E, CDK2, and CDK4 (Saini and Sanyal, 2015). Moreover, this pigment mediated apoptosis through the p53 activation pathway. C-phycocyanin also promoted antiproliferation by restraining PCNA expression and reduced cell survival via inhibiting NFκB (p65). C-phycocyanin demonstrated an anti-angiogenic role in cancer model by inhibiting cyclooxygenase-2 (Saini and Sanyal, 2014). Furthermore, it exerts antineoplastic effects via the regulation of membrane properties, raising calpain-9 and PPARγ expression while suppressing Wnt/β-catenin signaling (Saini and Sanyal, 2014).

C-phycocyanin was also reported to be efficient against mouth tumor in long-term smokers. In rodents treated with a combination of *Arthrospira* and *Dunaliella* extracts, the supplementation prevented the mouth-tumor development (Schwartz et al., 1988). Mathew et al. (1995) have evaluated the chemopreventive activity of *A. fusiformis* in reversing oral leukoplakia in pan-tobacco chewers in Kerala, India. Complete regression of lesions was observed in 45% evaluable subjects supplemented with “Spirulina,” as opposed to 7% in the placebo arm (Mathew et al., 1995). The aldehydes and ketones from tobacco smoke activate NADPH oxidase complexes in vascular tissues and in the lungs. Phycocyanin mimics cell intracellular bilirubin's role as an inhibitor of NADPH oxidase activity (McCarty et al., 2015), what makes it a promising agent to mitigate the pro-oxidative effects of smoke aldehydes and ketones. In turn, methyl gamma linoleate from *A. platensis* appeared to be a potent cytotoxic agent against human lung carcinoma—A549 cell line (Jubie et al., 2015).

Phycocyanin significantly decreases HeLa cells multiplication in comparison to control cells (Li et al., 2006, 2013; Yang et al., 2014). Furthermore, it activates the apoptotic pathway, including cell shrinkage in HeLa cell lines. Beaten cancer cells are enfeebled and incapable of rejuvenation. Ouhtit et al. (2014) confirmed that an *A. platensis* treatment of DMBA-induced rat mammary tumor decreased the cancer incidence from 87 to 13%. Immunohistochemical analysis revealed that supplementation with “Spirulina” reduced the expression of Ki-67 and estrogen α. *In vitro* studies showed that a “Spirulina” treatment inhibited cell proliferation, increased p53 expression, followed by the increased expression of its downstream target gene, Cdkn1a [alias p21 or p21(Waf1/Cip1)]. *A. platensis* increases Bax and decreases Bcl-2 expression, indicating the induction of apoptosis also 48 h after treatment.

Pan et al. (2015) showed the inhibitory effect of phycocyanin on the proliferation of the ovarian cancer SKOV-3 cell line. Phycocyanin also induced the apoptosis by the mitochondrial pathway.

The oral treatment with *A. maxima* performed by Chamorro-Cevallos et al. (2014) significantly reduced the detrimental effect of benzo[α]pyrene on the quality of mouse semen, protected from B[α]P-induced pre- and post-implant losses in the male dominant lethal test, and from B[α]P-induced post-implantation losses in treated females. Those results demonstrate the protective effects of *A. maxima* against B[α]P-induced mutagenicity in germ cells.

Antioxidant and anti-inflammatory effects of *A. platensis* have been shown in UVB-induced skin carcinogenesis in animal models. Investigators used wild-type and Ogg1 knockout-(KO) mice, as the absence of the enzyme produced by this gene raises the tumor incidence in response to UVB exposure. Dietary supplementation with *A. platensis* suppressed cancer development in both genotypes (Yogianti et al., 2014). Calcium spirulan from *Arthrospira* exhibited valuable antitumor characteristics, it inhibited the invasion of B16-BL6 melanoma, Colon 26 M3.1 carcinoma, and HT-1080 fibrosarcoma cells through reconstituted basement membrane (Mishima et al., 1998).

*In vitro* studies indicated that phycocyanin is able to diminish proliferation of leukemia cell line K-562 about 49% (Subhashini et al., 2004) and even 50% in case of hepatocellular carcinoma (liver cancer cell lines) S- and R-HepG2 cells (Roy et al., 2007).

Sometimes, better results are obtained when the compounds are used in combination. The mixing of phycocyanin with selenium demonstrates antiproliferative activity against melanoma A375 cells and human breast adenocarcinoma MCF-7 cells (Chen and Wong, 2008). A combination of selenium and “Spirulina” was also patented as the anticancer delivery vehicle (Riva and Oreal, 2013).

### Cardiovascular system protection

*Arthrospira* extracts and compounds may play an important role in the prevention of cardiovascular diseases. They lower the blood pressure, plasma lipid concentration, especially triacylglycerols. They indirectly modify total cholesterol and HDL/LDL (high- to low-density lipoprotein rates). Animal studies indicate that *Arthrospira* could be a novel preventive tonic for the heart and whole cardiovascular system. Nevertheless, human trials are still too small to prove this effect. All animal studies, preclinical and clinical trials are clearly reviewed by Deng and Chow (2010) in *Cardiovascular Therapeutics*.

“Spirulina” may be a prominent dietary supplement for patients with ischemic heart disease as it improves patients' lipid profiles and helps to lower blood pressure (Torres-Duran et al., 2007). In rodents, *Arthrospira* can have a protective effect on doxorubicin-related cardiac side effects (Khan M. et al., 2005; Khan et al., 2006) as well as be protective against heart attacks in humans (McCarty, 2010). Water extracts lower the fat absorption by inhibiting pancreatic lipase activity (Han et al., 2006).

Phycocyanin from “Spirulina” has a high bile binding capacity, lowers cholesterol solubility and intake by Caco-2 intestinal cells (in comparison to casein) (Nagaoka et al., 2005). In mice, C-phycocyanin can promote the CD59 gene expression. This activity prevents smooth muscle cell proliferation and the apoptosis of endothelial cells, reducing blood fat levels. In consequence, C-phycocyanin inhibits the development of atherosclerosis (Li et al., 2013). The GLA present in *Arthrospira* reduces cholesterol accumulation in the hypolipidemic nephrotic syndrome (Samuels et al., 2002).

### Biomaterials and bioactive substances from *Arthrospira* in tissue engineering

Polyhydroxyalkanoates (PHAs) are biodegradable and biocompatible polymers for tissue or organ scaffold construction in regenerative medicine. They can be extracted from different microorganisms including *Arthrospira* (Jau et al., 2005). Replacement of commercially available PHA biopolymers with equivalents obtained from *Arthrospira* or/and containing its biomass significantly increases the eucaryotic cell proliferation and decreases the risk of transplant rejection (de Morais et al., 2014). Since 2007, de Morais et al. (2010) have been studying the development of nanofibers produced from PLA (polylactic acid), polyethylene oxide and PHB (polyhydroxybutyrate) extracted from *Arthrospira* LEB 18 strain and incorporation of its compounds in artificial extracellular matrices. Their observations reveal that addition of LEB 18 biomass increases nanofibers conductivity (de Morais et al., 2010). PHB nanofibers from LEB 18 have higher mechanical durability with enhanced elasticity, tensile strength, and breaking elongation. Those characteristics support nutrient, growth factors, and metabolism byproducts distribution (de Morais et al., 2014).

A study carried out to compare poly-D,L-lactic acid (PDLLA) associated with LEB 18 biomass with classic PDDLA in the animal model of skin injury disclosed that PDDLLA/Sp scaffolds were more moldable and had better adherence to the wound (Steffens et al., 2014). The same authors showed that PDDLA/Sp increased cells viability in comparison to simple PDDLA matrices (Steffens et al., 2013). Formerly described antimicrobial, anti-inflammatory and immunostimulatory effects of biocompounds from *Arthrospira* provide additional benefits in biomatrices produced from cyanobacteria. They promote natural wound healing processes and minimize the risk of infection (de Morais et al., 2014).

### Other effects and perspectives

Some observed beneficial effects of *Arthrospira* supplementation cannot be classified, but their existence underlines the multiple therapeutical possibilities and how to take advantage of those unusual cyanobacteria.

Studies worth mentioning show that *Arthrospira* promotes the growth of probiotic microbiota (Parada et al., 1998; Kordowska-Wiater et al., 2011) from human gut, thus boosting the production of B6 vitamin that aids in energy release. This fact and its high content of rhamnose, glycogen, and GLA, which also participate in energy release, could predestine *Arthrospira* to be a potential medication in chronic fatigue syndrome. Notwithstanding, *Arthrospira* activity in four N-of-1 double-blind, randomized trials is comparable to placebo (Baicus and Baicus, 2007).

*Arthrospira* can turn out to be useful for aging-related diseases. A study performed on rats suggests it enhances expression level of the beta-adrenergic receptor in brain tissue, which decreases with age and is connected with dementia in elderly people (Gemma et al., 2002). Another animal study demonstrates “Spirulina”'s ability to protect neurons in the α-synuclein model of Parkinson Disease (Pabon et al., 2012). In rodents, it also exhibits protective effects against drug-related serious renal failure (Kuhad et al., 2006a,b; Lim et al., 2012). “Spirulina” also may have a particular utility in mitigating adverse effects of alcohol consumption thanks to its inhibitory effect on NADPH oxidase and certain nutraceuticals, including taurine, pantethine, and lipoic acid that may have a potential to boost conversion of acetaldehyde to acetate (McCarty, 2013). Due to the good ability to accumulate metal ions from solutions, *Arthrospira* can be a prominent source of therapeutical nanoparticles. The assimilation process is pH-independent and may be a part of the biofunctionalized (antibacterial) Au nanoparticles biosynthesis in *Arthrospira* platensis (Savvaidis, 1998; Suganya et al., 2015).

### *Arthrospira* formulations as cosmetics

The abundance of natural bioactive compounds makes *Arthrospira* extracts perfect for use in commercial as well homemade cosmetics. Formulas containing “Spirulina” are mostly sold as anti-aging products that combat the action of free radicals, provide hydration, and protection to the skin (e.g., Patent EP2695604 A1^3^). Due to its antimicrobial activity, cosmetics against acne, and other bacterial skin infections are enriched with “Spirulina” extracts (W&P Cosmetics)^4^.

### Cyanobacteria from the *Arthrospira* genus in animal diet supplementation

Previously, animals were fed with *Arthrospira* to examine the effect on a living organism, which was a preliminary model for human nutrition (Fevrier and Seve, 1975). Nowadays, “Spirulina” is treated as a valuable supplement that supports animal well-being and their products quality.

Dietary “Spirulina” supplementation in poultry influences both the yellowness and redness of broiler flesh (Toyomizu et al., 2001b), increases several immunological functions, rising resistance to infections (Qureshi et al., 1996; Al-Batshan et al., 2001) and could be as effective as the diet with synthetic pigment in producing an agreeable egg yolk color (Zahroojian et al., 2011).

Saeid et al. (2013) performed studies focused on the effect of supplementation of *A. maxima* enriched with Cu on the production performance, metabolism and physiological parameters in fattening pigs. Examination revealed that supplementation with “Spirulina” extract reduced the liver toxic stress and total cholesterol levels by 10%, rose the glucose level in blood, which may indicate a better energy metabolism and caused a more intense red color of the meat (Saeid et al., 2013).

## Conclusions

Recent progress in genomics and molecular biology allowed for a better understanding of extraordinary microorganisms—the edible cyanobacteria from the *Arthrospira* genus. Phylogenetic analyses and bioinformatic approaches shed a new light on genomes' structure and obstacles in genetic engineering. Genetic analyses identified numerous genes responsible for hydrogen production, stress adaptation, restriction-modification systems, and others, unraveling the processes sustaining the functions of the organisms. Genome and proteome studies have proven the absence of toxicity factors, supporting the idea that Arthrospira products are safe to consume. Studies conducted for many years showed that *Arthrospira* was an organism extremely resistant to environmental stresses. As a rich source of macro- and microelements, vitamins, pigments, protein, polysaccharides, GLA, and other bioactive compounds, it has become a vivid object of interest in food and health industry. Increased interest in this organism results in higher demand for biomass production. Therefore, multiple studies aiming at improving its cultivation conditions and lowering the costs were carried on. *Arthrospira* was also proven to be very valuable from the medical point of view. Numerous researches conducted on animal models and patients confirmed its immunomodulatory properties, which is strictly connected with antitumor activity against different types of lesions. Apart from empowering human immunological response, chemical compounds extracted from *Arthrospira* exhibits antibacterial, antifungal, and antiviral activity. Interestingly, the probable mechanism of action against enveloped viruses is so universal that “Spirulina” extracts inhibit internalization and replication of potentially every enveloped virus, including HIV-1 and HCV. Fatty acids, especially GLA provide a protective effect for the cardiovascular system, regulating cholesterol levels. A recent development of modern medicine—tissue engineering—can take advantage of *Arthrospira* polysaccharides used for scaffold construction in tissue/organ regeneration process. The addition of biocompounds from *Arthrospira* enhances stem cells proliferation and promotes wound healing by supporting the immunological system and providing nourishing substances. Other valuable effects worth mentioning include the promotion of human gut microflora growth, clearance from different types of toxins and neuron anti-aging properties.

## Author contributions

The authors of the general work conception are KW and MW. Reviewers and actual authors of the manuscript text are MAF (genetic section), AM (physiology), MDF (medicine). Critical revision for important intelectual content: AW, KW, and MW. Proofreading, edition, and final approval of the version to be published was made by all authors mentioned above. All questions should be addressed to KW.

### Conflict of interest statement

The authors declare that the research was conducted in the absence of any commercial or financial relationships that could be construed as a potential conflict of interest.

## Footnotes

^1^World Health Organization (1993), Geneva, Switzerland.

^2^Oxford Dictionary (2017). Available online at: https://en.oxforddictionaries.com/definition/superfood (Accessed February 16, 2017).

^3^Patent EP2695604A1—*Spirulina-Containing Cosmetic Composition and Cosmetic Treatment Method* (2017). Google Books. Available online at: http://www.google.com/patents/EP2695604A1?cl=en#backward-citations (Accessed January 24, 2017).

^4^W&P Cosmetics (2016). Available online at: http://wp-cosmetics.pl/ (Accessed *February* 16, 2017).
